# Characteristics and motivational factors for joining a lay responder system dispatch to out-of-hospital cardiac arrests

**DOI:** 10.1186/s13049-022-01009-1

**Published:** 2022-03-24

**Authors:** A. Högstedt, M. Thuccani, E. Carlström, A. Claesson, A. Bremer, A. Ravn-Fischer, E. Berglund, M. Ringh, J. Hollenberg, J. Herlitz, A. Rawshani, P. Lundgren

**Affiliations:** 1 Prehospen – Centre for Prehospital Research, Faculty of Caring Science, Work Life and Social Welfare, University of Borås, Borås, Sweden; 2 Department of Molecular and Clinical Medicine, Institute of Medicine, Sahlgrenska Academy, University of Gothenburg, Gothenburg, Sweden; 3 Institute of Healthcare Sciences, Sahlgrenska Academy, Gothenburg University, Gothenburg, Sweden; 4 USN School of Business, University of South-Eastern Norway, Kongsberg, Norway; 5 Department of Clinical Science and Education, Södersjukhuset, Karolinska Institutet, Stockholm, Sweden; 6 Faculty of Health and Life Sciences, Linnaeus University, Växjö, Sweden; 7 Department of Cardiology, Sahlgrenska University Hospital, Gothenburg, Sweden

**Keywords:** Volunteer, Lay volunteer, Lay responder, OHCA, Bystander, CPR, AED, Motivation, Self-determination theory

## Abstract

**Background:**

There has been in increase in the use of systems for organizing lay responders for suspected out-of-hospital cardiac arrests (OHCAs) dispatch using smartphone-based technology. The purpose is to increase survival rates; however, such systems are dependent on people’s commitment to becoming a lay responder. Knowledge about the characteristics of such volunteers and their motivational factors is lacking. Therefore, we explored characteristics and quantified the underlying motivational factors for joining a smartphone-based cardiopulmonary resuscitation (CPR) lay responder system.

**Methods:**

In this descriptive cross-sectional study, 800 consecutively recruited lay responders in a smartphone-based mobile positioning first-responder system (SMS-lifesavers) were surveyed. Data on characteristics and motivational factors were collected, the latter through a modified version of the validated survey “Volunteer Motivation Inventory” (VMI). The statements in the VMI, ranked on a Likert scale (1–5), corresponded to(a) intrinsic (an inner belief of doing good for others) or (b) extrinsic (earning some kind of reward from the act) motivational factors.

**Results:**

A total of 461 participants were included in the final analysis. Among respondents, 59% were women, 48% between 25 and 39 years of age, 37% worked within health care, and 66% had undergone post-secondary school. The most common way (44%) to learn about the lay responder system was from a CPR instructor. A majority (77%) had undergone CPR training at their workplace. In terms of motivation, where higher scores reflect greater importance to the participant, intrinsic factors scored highest, represented by the category *values* (mean 3.97) followed by extrinsic categories *reciprocity* (mean 3.88) and *self-esteem* (mean 3.22).

**Conclusion:**

This study indicates that motivation to join a first responder system mainly depends on intrinsic factors, i.e. an inner belief of doing good, but there are also extrinsic factors, such as earning some kind of reward from the act, to consider. Focusing information campaigns on intrinsic factors may be the most important factor for successful recruitment. When implementing a smartphone-based lay responder system, CPR instructors, as a main information source to potential lay responders, as well as the workplace, are crucial for successful recruitment.

**Supplementary Information:**

The online version contains supplementary material available at 10.1186/s13049-022-01009-1.

## Background

Dispatching organized lay volunteers as first -responders (in this article named termed lay responders) in suspected out-of-hospital cardiac arrest (OHCA), hasve been shown to increase the rate of cardiopulmonary resuscitation (CPR) before emergency medical services (EMS) arrives on scene [1–4]. Several lay responder systems have been implemented worldwide aiming to decrease the time to treatment in suspected OHCA [3, 5–7]. Both American Heart Association (AHA) Guidelines 2020 and European Resuscitation Council (ERC) Guidelines, acknowledge the importance of using technology to engage communities and alert first responders to suspected cardiac arrests through smartphone apps or text messages [8, 9].

Technical systems in which lay responders, either as off-duty first responders or civilians trained in CPR, are activated by Emergency Dispatch Centers (EDC) by means of a smartphone application, are a prerequisite for coordinated action between dispatch and lay responders. There are several systems in different parts of the world [3, 5, 7, 10]. The foundation of these systems is the commitment from lay responders. Today, knowledge about the motivation for joining a lay responder system is lacking. The objectives of this study were to explore the characteristics of lay responders and to describe their underlying motivational factors for joining a CPR lay responder system.

## Methods

### Design, settings and subjects

The study design was descriptive and cross-sectional. A modified version of a validated survey, the Volunteer Motivation Inventory (VMI) [11], was used to quantify the motivation factors behind joining as a volunteer in a lay responder system answering to suspected OHCAs and to describe the characteristics of the lay responders.

The study was conducted in the region of Västra Götaland, a geographical and administrative region in the southwest part of Sweden with a population of about 1.7 million people. The region represents both urban areas, including the city of Gothenburg, and rural areas, including more than 100 inhabited islands. The mean population density per square kilometer in the region of Västra Götaland is 72.9, compared to 25.5 in Sweden overall and ranges from 6.6 to 1302 [12, 13]. A lay responder system (SMS-lifesaver) was introduced in October 2016. The requirements for signing up as a lay responder was being age 18 years or older and having completed a CPR course within the last three years. The SMS-lifesaver system is dispatched by EDC to lay responders in close vicinity of the OHCA victim. The task is either to obtain a public automated external defibrillator (AED) or to proceed to the victim to perform CPR. The lay responder can approve or decline the alert and may pause the lay responder system at any time, if needed. Information about the lay responder system and recruiting campaigns were spread on a political and community level through council meetings. Volunteer organizations and well-known associations, collaboration partners, and larger companies, together with more traditional first responders in medical life support alerts such as police and fire services, were invited in the implementing process to spread information within their own organizations. Information was also spread through social media, newspapers, and through radio about six months ahead of start and then continuously during the first year of service implementation. By the time of the study, approximately 8,000 lay responders had been recruited in the region and when signing up to the system, the lay responders also agreed to be part of a research project. When responding to the survey, they also gave their consent to participate.

All lay responders recruited to the lay responder system during a three-month period, from 1 June 2017 to 29 September 2017, were included in the study. By that time, the lay responder system had then been running for eight months in the region.

### Ethics approval and consent to participate (Appendix 1)

The study was approved by the Swedish Ethical Review Authority (No 2019-05305 and No 2016/1531-31/4).

### Data collection

Google Forms was used for distributing the survey. A web link was sent out to the subjects’ smartphones twice with a five-day interval and twice as an email. Between each distribution, lay responders that had answered the survey were cleared from the register before the next distribution.

Data on registered lay responders were collected from the lay responder register. The information provided ahead of the study was gender, year of birth, phone number, email, and recruitment day.

### Survey

The VMI survey (Additional file 1) is based on Self Determination Theory (SDT) [11]. In this theory, “motivation” is explained with the perspective that people volunteer in order to please one or more needs or motives [14–16]. The basis of the theory is the disparity between intrinsic and extrinsic motivation. Intrinsic motivation comes from within; it is defined as interesting and enjoyable for the volunteer and includes core values such as compassion, respect, commitment, and a personal sense of morality. In contrast, extrinsic motivation is based on gaining something that is separable from the act, e.g., it may imply avoiding punishment, gaining payment, or gaining self-esteem [17]. The same act can serve disparate functions for the volunteer [18]. The VMI is designed by Esmond and Dunlop [11] and based on ten categories describing volunteer motivation [19, 20]. The categories are related to intrinsic or extrinsic motivation or both [16]. The categories are *values,* which is the only category representing pure intrinsic motivation, as well as *understanding, career development, social, self-esteem*, *protective, reciprocity, recognition, reactivity* and *social interaction,* being extrinsic on different levels (Additional file 2).

The original VMI survey has been validated on a total of 2,444 volunteers from 15 different organizations in one of the largest studies of volunteer motivation. The original VMI survey contains 44 statements about the respondents’ experiences and motivation. Every statement requires an answer on a Likert scale from 1 to 5 with 1 being “strongly disagree” and 5 being “strongly agree”. Each statement is categorized into the motivational categories, by the VMI template (Additional file 2). The higher score of a category, the more impact this category has on motivation and thereby also on self-determination of the volunteer.

In this study, the original survey was modified by the authors to suit lay responders acting on unplanned short notice (Additional file 3), and then approved by the originators (Additional file 4).

To ensure readability and validity, the original English version was translated to Swedish and then back translated to English by a professional translator. The Swedish version and the second English version were validated with a “Content Validity Index” (CVI) [21]. For characteristics of the lay responders, questions about age, gender, education, and work, were added to the VMI survey. Definitions of education and age clusters were taken from official Swedish governmental statistics [12].

### Statistical analysis

The results of the questionnaire were decoded using the VMI template, modified to our shortened version.

The highest score reflected the type of motivation that had the greatest importance to the lay responder. Mean values for each question were calculated together with standard deviation (SD). The different questions were then categorized according to the motivational categories.

Characteristics of lay responders are summarized; categorical variables are presented in percentages and continuous variables are presented in mean and standard deviation. Occupational area was subdivided with a subgroup called *other occupations* when only a total of ≤ 3 subjects had reported having the same occupation. Volunteers in the subgroup *other occupations* were additionally summarized in a separate table in more detail. Data was stratified based on gender, age, and level of education, with the purpose of examining if these characteristics were associated with certain motivations.

All analysis was considered exploratory and comparisons are made by assessing patterns instead of performing hypothesis tests.

The Excel software version 16.0.12904.35902 was used for calculations of data. Cluster analysis and further computations were performed in R (v. 4.0.3, R Foundation for Statistical Computing).

## Results

The survey was sent out to 800 subjects and out of them a total of 461 subjects completed the survey, resulting in a 58% response rate.

### Characteristics of the lay responders

Lay responder characteristics are shown in Table 1. Out of the 461 lay responders that completed the survey, 58% were women, (total survey population 59%). There was a higher representation of women within all age subgroups except within the age group 55–64, in which 42% were women (total survey population 44%). Most lay responders had a high level of education, (66% post-secondary education). The most common occupational area was *healthcare* (36%). CPR was learned mostly at work (77%), and in schools (16%). Lay responders learned about the lay responder system mainly through their CPR instructor (44%), followed by work (20%) and friends and family (18%).

**Table 1:** Lay volunteer characteristics

Characteristic	n = 461 (%)		
Female	269 (58.3)		
*Age and sex*			
18–24	66 (14.3)		
Female	46 (70)		
25–39	209 (45.3)		
Female	121 (57.9)		
40–54	134 (29.1)		
Female	80 (59.7)		
55–64	41 (8.9)		
Female	17 (41.5)		
65–74	9 (2.0)		
Female	4 (44.4)		
75 or older	2 (0.4)		
Female	1 (0.2)		
*Occupational area*			
Healthcare	168 (36.4)		
Student	71 (15.4)		
Police- or fire service	43 (9.3)		
Teacher	23 (5.0)		
EMS	13 (2.8)		
Prison guard/watchman	12 (2.6)		
Retired	11 (2.4)		
Engineer	5 (1.1)		
Other occupations, 3 or less of the same	115 (24.9)		
*Education*		*Grouped in cluster analysis*	
< 9 years	1 (0.2)	Primary education	
Primary school ≥ 9 years	11 (2.4)	Primary education	12 (2.6)
Secondary school, ≤ 2 years	35 (7.6)	Secondary education	
Secondary school, 3 years	111 (24.1)	Secondary education	146 (31.7)
Post-secondary, < 3 years	92 (20.0)	Post-secondary education	
Post-secondary, ≥ 3 years	202 (43.8)	Post-secondary education	
Postgraduate education	9 (2.0)	Post-secondary education	303 (65.7)
*Learned CPR through*			
Work	357 (77.4)		
School	74 (16.1)		
Organizations	15 (3.3)		
Unknown	7 (1.5)		
Fireservice	4 (0.9)		
Other, 3 or less of the same	4 (0.9)		
*Learned about the CPR volunteer first-responder system through*			
CPR-educator	203 (44.0)		
Work	92 (20.0)		
Friends and family	82 (17.8)		
Social media	64 (13.9)		
Article in newspaper	12 (2.6)		
Other	8 (1.7)		

### Motivation of the lay responders

The category most important for motivation (Table 2) and thereby the decision to become a lay responder was *values* (Mean (M) 3.97; Standard deviation (SD) 0.55) In the category *values,* the top scored statements were *“I am a SMS-lifesaver because I am concerned about those who suffer from cardiac arrest”* (M 4.78; SD 0.46) and *“I am a SMS-lifesaver because I feel it is important to help others”* (M 4.73; SD 0.54). The next most important category *reciprocity* (M 3.88; SD 0.90); the top scored question was*”I am a SMS-lifesaver because I believe that what goes around comes around”* (M 4.32; SD 0.96) and third most important category, *self-esteem* (M 3.22; 0.92), had as its top scored question “*I am a SMS-lifesaver because I feel that volunteering is a feel-good experience”* (M 4.35; SD 0.84). The least important category was *reactivity* (M 1.65; SD 0.79), where the top scored question was *“I am a SMS-lifesaver because I like to help people; I have been in difficult positions myself”* (M 2.06; SD 1.33).

**Table 2:** Motivation score based on motivational categories within the Self Determination Theory

Motivation category	Mean	SD
Values	3.97	0.55
Reciprocity	3.88	0.90
Self-esteem	3.22	0.92
Recognition	2.76	0.77
Understanding	2.74	1.08
Career development	2.22	0.90
Social	2.04	0.80
Social interaction	2.01	0.89
Protective	1.93	0.64
Reactivity	1.65	0.79
All	2.64	0.82

Higher score reflects motivation of greater importance to the participant, i.e. more self-determined, and the lower score represent motivation of less importance, i.e. less self-determined. Three is considered neutral

### Stratification of the lay responders

Stratification based on age (Additional file 5: Figure S1) showed that with increasing age, the mean scores decreased for the motivation categories *career development*, *self-esteem,* and *understanding*. Additionally, *career development, social interaction, understanding* and a few other motivation categories had a higher mean score in the older age groups 65–74 years and 75 years or older. However, the volunteers in these two age groups make up only 2.4% of the entire study population.

Stratifying based on level of education (Additional file 5: Figure S2) showed that *career development*, *recognition, self-esteem, social interaction, social, understanding* and *values* had higher mean scores for volunteers that had completed secondary education.

Stratifying based on gender showed no significant differences between the genders for each motivation category (Additional file 5: Figure S3).

## Discussion

This study aimed to explore characteristics and to quantify the underlying motivational factors for joining a CPR lay responder system. Our main findings were that CPR instructors were the most important information source to potential lay responders and even though healthcare personnel were the most represented occupation, lay responders came from diverse occupational backgrounds. Six out of ten lay volunteers in the study were women and the age group 25–39 was overrepresented among the total study population (45%). Motivation to join the lay responder system was not purely intrinsic or extrinsic, but intrinsic motivation, *values*, an inner belief in doing good for others, dominated. The most important extrinsic motivation categories were *reciprocity*, a belief that doing good for others will come back to the volunteer later on in life, and *self-esteem*, a way of increasing one’s own feelings of self-worth and *self-esteem.*

### Possible finding mechanisms

Most lay responders received their CPR education through their work. This may be due to the fact that CPR training has become a natural part of the working life in Sweden [22]. A large proportion of the lay responders have a background in healthcare, police, and fire services where CPR training is a repeated element, but a significant share of the investigated population has a variety of professions, which indicates that recruiting should aim towards the public in general. Healthcare personnel is the largest group of occupations and despite or because of the nature of their task assignments during work, they are motivated to become lay responders off duty. Most lay responders in the study, had a high level of education; 66% had post-secondary education compared with the national average of 44% in people 25–64 years of age [12], and students were the second largest group of occupation, which indicates that recruiting campaigns aiming at workplaces that require higher education or towards colleges and universities might be succesful.

Most lay responders learned about the lay responder system through their CPR instructor and if such participants were to be asked a direct question about joining a lay responder system, recruitment might be additionally successful. This corresponds well with Busell and Forbes’s [23] study that people are four times as likely to volunteer when they received a targeted question than when they were not. Education initiatives vary around the world; CPR instructors can for example be a teacher, ambulance personnel, students, or a family member [24]. Apart from CPR instructors, participants in this study learned about the lay responder system through friends, family, and work colleagues. Social media, in this case information and storytelling through Facebook [25], had five times more impact on recruiting than more traditional media such as articles in newspapers, indicating that campaigns in social media can play an important role when recruiting lay responders.

*Values* as the most important motivational category for volunteering is consistent with previous studies, including the study by Esmond and Dunlop [11, 26–30]. Appealing to intrinsic and altruistic motivational factors as *values*, emphasizing the importance of lay responder action for successful resuscitation in OHCA, might therefore be of significant importance when recruiting lay responders.

*Reciprocity,* being the second most important category, also corresponds with the result in the study by Esmond and Dunlop [11]. In our study, the category is most important among the group *other occupations* and is both intrinsic and extrinsic. The tagline “the effort you put in today will bring good things later on in life” could be used in recruitment campaigns, thus reinforcing the idea that if you expect help when in need, you also need to be a helper.

The category *self-esteem* seems to be more important for motivation and determination for volunteering in our study than in the Esmond and Dunlop survey [11], which might be explained by the fact that the project has run great interest and that the commitment is a lifesaving act. Phung et al. [29], state that self-esteem and personal satisfaction from helping others were an important factor for motivation for becoming and remaining a lay responder (defined in that study as community first responder, or CFR). The feeling of meaningfulness of being a part of a lay responder system ought to be strong, as the act of CPR or bringing an AED to a patient can make the difference between life and death. It corresponds also with Grouzet et al. [31], arguing that people want to feel effective and have a feeling of mastering their world by searching for optimal challenging activities.

*Recognition* was more important for motivation in the Esmond and Dunlop survey [11], which may be due to the fact that lay responder systems require less contribution in terms of time and effort.

Phung et al., also identified the lay responder engagement as a route towards a future career as a health professional [29]. In our study, *career development* scored low and that might be explained by the differences in setting, where most CRFs also are trained to respond to other emergency calls than OHCAs and thereby gain more professional contacts and a better insight to health professions and EMS than most lay responders in our study. Barry et al. [28] also identified lay responders that were motivated based on past experiences. That corresponds with the category *reactivity,* which scored lowest among the motivational categories in our study. This might be explained by the fact that inclusion criteria in the Barry et al. study was to have personal experience of OHCA situations.

The overall lower scores among the lay responders in our study compared to the Esmond and Dunlop study [11] could be explained by the reason that a longer commitment requires a higher level of motivation.

Most studies on motivation among lay responders acting on OHCAs investigated community first responders (CFRs). Motivation based on intrinsic motivational factors, *values,* and extrinsic motivational factors, *self-esteem,* correspond well with the motivational factors for the lay responders in our study. Past experiences (*reactivity*) and *career development* are factors that appear more important as motivation for CRFs than lay responders in our study. This could be explained by the different settings of the lay responders. Most members of CRF groups plan their participation ahead of time and work on a schedule. They sometimes work in pairs, are trained in basic life support, and are handed a dedicated alert phone that receives a limited range of dispatched emergency calls. Also, most CFR groups work in rural areas [29, 30]. The subjects in our study participate in the dispatched EMS chain by being alerted to their personal mobile phone, if they happened to be in the geographic area where a suspected OHCA occurred, and they represent both rural and more densely populated areas. Hence, their actions are not planned ahead and could take place at any time.

### Stratification findings

The decreasing mean scores for *career development* with each increasing age group in the stratification based on age (Additional file 5: Figure S1) suggests that this motivation becomes less important with increasing age. Note that the mean scores for certain motivational categories such as *career development* and *understanding* where higher for the oldest age groups, 65 years and older. These results must be interpreted with caution as these volunteers only made up 2.4% of the study population. Intuitively, young volunteers that are either students or in the early stages of their careers are likely to value the experience that volunteering will yield their careers. A similar pattern for *career development* was presented in Ho, You and Fung’s research [32]; the authors described that younger volunteers were more motivated to improve their future employment prospects than older volunteers. Additionally, greater importance for *understanding* in the younger volunteers suggests that these volunteers are motivated to learn through the experiences from volunteering. A large proportion of the volunteers were also healthcare professionals. Therefore, *career development* and *understanding* are likely strong motivators amongst the younger volunteers with careers in healthcare.

*Self-esteem* was also a stronger motivator for younger volunteers which corresponds with Cho, Bonn and Han’s research [33]. These results suggest that younger volunteers are in part motivated to become first responders with the expectation that the experience will elevate their self-worth. Here again, the higher means score of the age group 65–74 years is possibly misleading.

*Social interaction* was valued more in the older age groups. However, there were very few volunteers in these age groups, thus these results may not be representative of a greater population.

### Practical implications

Most patients who suffer from OHCA do not survive [34, 35]. The chance to survive increases with fast response, early treatment with CPR and the use of an AED [36, 37]. However, in the last 30 years, EMS systems response times have increased [22]. Thus, involving lay responders could be an important part in efforts to increase survival rates and is recommended both by AHA and ERC.

As CPR instructors seemed to be the most common recruiter of lay responders, focus and support especially in regards to CPR instructors should be a priority when recruiting volunteers to a lay responder system. It is also important that CPR instructors have knowledge about their important role as recruiters and what motivates lay responders to join a lay responder system in order to increase participation. A mandatory question about participation to a lay responder system might be a successful recruitment strategy.

Additionally, this study showed that the lay responders are largely represented by younger individuals in the age group 25–39 years. Therefore, information campaigns through social media could be effective in recruiting younger responders; this corresponds with the annual report on the Internet and social media usage in the Swedish population [38]. However, an examination of the subjects with *other occupations* showed that CPR training through work and the CPR instructor were effective recruitment channels. This further confirms the CPR instructors’ crucial role in recruitment. Thus, information campaigns in social media can likely be an additional tool useful to an already successful concept.

Though the importance of different motivation categories differed among the clusters, all clusters showed that motivation categories with largely intrinsic components, e.g., *values* and *reciprocity*, had the greatest impact on motivation for the lay responders. Highly valued motivations with largely extrinsic components, e.g., *reactivity,* did not exist, and that suggests that lay responders are less likely to have extrinsic factors as their motivation. This implies that focusing information campaigns on intrinsic factors could be important for successful recruitment. This could be exemplified by the difference a lay responder in close vicinity to an OHCA victim can make combined with real-life-stories of OHCA survivors that may increase motivation, self-determination, and commitment.

Among the statements in the survey, “*Being appreciated by the organization behind SMS-lifesavers is important to me*” scored above neutral (M 3.56; SD 1.15) with 39% of the population responding *agree* or *strongly agree*, which suggests that there is a considerable group of lay responders that need some type of feedback and attention. Providing *recognition* for skills and contribution is a major challenge for managers of lay responders in a lay responder system. The result of the survey identifies the need for finding ways to formally and perhaps informally recognize and reward lay responders. One way to address *recognition* could be by providing a small stipend [39] or a thank you note [40]. Another way for providing recognition could be providing an object that can be made visible to others, like a bumper sticker or a keychain, when joining the lay responder system.

An interesting finding from the cluster analysis was that clusters three and four were smaller groups and had a different ranking of the motivation categories. The level of education differed the most between the clusters, with a majority of the volunteers in clusters three and four who had completed secondary education, whilst clusters one and two had mostly completed post-secondary education. Thus, level of education may have an association with motivation, which in turn can be important for the recruitment of more volunteers.

In order to better understand motivation behind volunteering to a lay responder system such as SMS-lifesavers, studies that investigate e.g. whether motivation changes over time and/or according to the frequency of alerts, and what would motivate those with CPR knowledge who not respond to recruiting campaigns, would be of benefit in the future, both quantitatively and qualitatively.

### Limitations

The survey was distributed to a relatively small group of lay responders in the western part of Sweden and the result might differ from other regions and countries, although the region includes both urban and rural areas. The response rate was 58%, which may affect the validity of the results; however, mean response rate in similar studies are between 43–53% and survey response rates have generally declined over the past years [41, 42]. General characteristics such as gender and age do not differ significantly between the total population and the study cohort; however, motivation might diverge between the two groups.

To our knowledge, Esmond and Dunlop’s [11] research is the largest study of volunteer motivations. The VMI is validated, but the scoring guide and methodology have some weaknesses. Translating answers that are not equidistant to a Likert like scale, even with ordinal answers, only indicates trends in the result, not an entirely true number.

## Conclusion

This study indicates that motivation to join a first responder system mainly depends on intrinsic factors, an inner belief of doing good, but there are also extrinsic factors, i.e. earning some kind of reward from the act, to consider. Focusing information campaigns on intrinsic factors may be the most important factor for successful recruitment. When implementing a smartphone-based lay responder system, CPR instructors, as a main information source to potential lay responders as well as the workplace, are crucial for successful recruitment.

## Supplementary Information

**Additional file 1**. Volunteer Motivation Inventory (VMI) survey.

**Additional file 2**. Definition of categories - description of results.

**Additional file 3**. VMI Template.

**Additional file 4**. Approval from the originators.

**Additional file 5**. Cluster analysis.

## Data Availability

The datasets generated and analyzed during the current study are not publicly available due to the fact that they contain information that could compromise the privacy of research participants. Data that support the findings are available via the corresponding author upon reasonable request.
