# Supplementary material for: Characteristics and motivational factors for joining a lay responder system dispatch to out-of-hospital cardiac arrests
Source: Scand J Trauma Resusc Emerg Med. 2022 Mar 24;30:22. doi: 10.1186/s13049-022-01009-1 (PMC8943963; doi:10.1186/s13049-022-01009-1)
Supplement: Supplementary file 1 — Additional file 1. Volunteer Motivation Inventory (VMI) survey. [file 13049_2022_1009_MOESM1_ESM.pdf]

Supplement 1

Volunteer Motivation Inventory (VMI) survey related to categories – numbers from the original survey to fit scoring guide (Supplement 2)

1	Values (Va)	I am a SMS-lifesaver because I am concerned about those who suffer from sudden cardiac arrest.
2	Recognition (Rn)	Being appreciated by the SMS-lifesaver agency is important to me.
4	Reciprocity (Rp)	I am a SMS-lifesaver because I believe that you receive what you put out in the world.
5	Reactivity (Rc)	I am a SMS-lifesaver because I like to help people, I have been in difficult positions myself.
6	Self-Esteem (SE)	I am a SMS-lifesaver because I feel that volunteering is a feel-good experience.
7	Social (So)	I am a SMS-lifesaver because my friends also are SMS-lifesavers.
8	Career Development (CD)	I am a SMS-lifesaver because it gives me an insight in the professions, I am interested in.
10	Protective (Pr)	I am a SMS-lifesaver because it gives good conscience helping people in vulnerable situations.
11	Values	I am a SMS-lifesaver because I am genuinely care for the topic cardiac arrest.
12	Recognition	Being appreciated by EMS-staff and other SMS-lifesavers is important to me.
13	Social Interaction	It is important to me that people around me know that I am a SMS-lifesaver.
14	Reactivity	Being a SMS-lifesaver gives me a chance to try to ensure people do not have to go through what I went through.
15	Self-Esteem	I am a SMS-lifesaver because it makes me feel like a good person.
16	Social	I am a SMS-lifesaver because people I’m close to are.
18	Understanding (Un)	I am a SMS-lifesaver because volunteering allows me to gain a new perspective on things.
19	Protective	I am a SMS-lifesaver because volunteering helps me work through my own personal problems.
20	Values	I am a SMS-lifesaver because I feel compassion towards people suffering from cardiac arrests and their relatives.
21*	Recognition	I do not need feedback after a SMS-lifesaver mission.

22	Social Interaction	I am a SMS-lifesaver because I feel that volunteering is a way to build one’s social networks.
23	Reactivity	I often relate to me being a SMS-lifesaver in my own personal life.
24	Self-Esteem	I am a SMS-lifesaver because it makes me feel important.
25	Social	I am a SMS-lifesaver because people I know share an interest in voluntary community service.
26	Career Development	I am a SMS-lifesaver because I feel that the experience will benefit job opportunities.
27	Understanding	I am a SMS-lifesaver because it lets me learn through direct hands-on experience.
28	Protective	I am a SMS-lifesaver because volunteering is a good escape from my own troubles.
29	Values	I am a SMS-lifesaver because I feel it is important to help others.
31	Social Interaction	Being a SMS-lifesaver provides an interesting topic when socializing new acquaintances.
32	Reactivity	Being a SMS-lifesaver helps me deal with some of my own experiences.
33	Self-Esteem	I am a SMS-lifesaver because it makes me feel useful.
34	Social	I am a SMS-lifesaver because others with whom I am close place a high value on such volunteering.
35	Career Development	I am a SMS-lifesaver because it gives me an opportunity to develop my skills.
36	Understanding	I am a SMS-lifesaver because I can learn how to deal with different kind of people.
37	Protective	I am a SMS-lifesaver because no matter how bad I am feeling, a mission helps me forget about it.
38	Values	I am a SMS-lifesaver because I can do something for a cause that is important to me.
39	Recognition	I feel that it is important to receive recognition for being a SMS-lifesaver.
40	Reciprocity	I am a SMS-lifesaver because I believe that what goes around comes around.
42	Social	I am a SMS-lifesaver because itis an important activity to the people I know best.
43	Understanding	I am a SMS-lifesaver because I can explore my own strengths.
44	Protective	I am a SMS-lifesaver because it makes me feel less lonely.
