# Supplementary material for: Characteristics and motivational factors for joining a lay responder system dispatch to out-of-hospital cardiac arrests
Source: Scand J Trauma Resusc Emerg Med. 2022 Mar 24;30:22. doi: 10.1186/s13049-022-01009-1 (PMC8943963; doi:10.1186/s13049-022-01009-1)
Supplement: Supplementary file 2 — Additional file 2. Definition of categories - description of results. [file 13049_2022_1009_MOESM2_ESM.pdf]

Supplement 2

Definition of categories – description of results

Following description is a quote from *Developing the Volunteer Motivation Inventory by* Esmond and Dunlop [11].

Values (Va) – Describes the situation where a volunteer is motivated by the prospect of being able to act on firmly held beliefs that it is important for one to help others. High scores on this scale suggest that a volunteer is motivated to help others just for the sake of helping. Low scores indicate that a volunteer is less interested in volunteering as a means of helping others (Clary, Snyder and Ridge, 1992).

Reciprocity (Rp) – Describes a situation where a volunteer enjoys volunteering and views it as a very equal exchange. The volunteer has a strong understanding of the 'higher good'. High scores on this scale indicate that the volunteer is motivated by the prospect that their volunteering work will bring about good things later on. Low scores indicate that the prospect of their volunteering work bringing about good things later on is not as important to them.

Self-Esteem (SE) – Describes a situation where a volunteer seeks to improve their own self esteem or feelings of self-worth through their volunteering. High scores on this scale indicate that a volunteer is motivated by the prospect of feeling better about themselves through volunteering. Low scores indicate that a volunteer does not regard volunteering as a means of improving their self-esteem.

Recognition (Rn) – Describes a situation where a volunteer enjoys the recognition that volunteering gives them. They enjoy their skills and contributions being recognised, and this is what motivates them to volunteer. High scores indicate a strong desire for formal recognition for their work, whereas low scores indicate a lesser level of interest in formal recognition for their volunteering work.

Understanding (Un) – Describes a situation where a volunteer is particularly interested in improving their understanding of themselves, or the people they are assisting and/or the organisation for which they are a volunteer. High scores on this scale indicate a strong desire to learn from their volunteering experiences. Low scores on this scale indicate a lesser desire of a volunteer to improve his or her understanding from their volunteer experience (Clary, Snyder and Ridge, 1992).

Career Development (CD) – Describes a situation where a volunteer is motivated to volunteer by the prospect of gaining experience and skills in the field that may eventually be beneficial in assisting them to find employment. High scores on this scale are indicative of a strong desire to gain experience valuable for future employment prospects and/or to make work connections. Low scores on this scale are indicative of a lesser interest in gaining experience for future employment or in making work connections.

Social (So) – Describes a situation where a volunteer seeks to conform to normative influences of significant others (e.g. friends or family). High scores on this scale indicate that the volunteer may be volunteering because they have many friends or family members who also volunteer, and they wish to 'follow suit'. Low scores may indicate that a volunteer has few friends or family members who already volunteer (Clary, Snyder and Ridge, 1992).

Social Interaction (SI) – Describes a situation where a volunteer particularly enjoys the social atmosphere of volunteering. They enjoy the opportunity to build social networks and interact

with other people. High scores indicate a strong desire to meet new people and make friends through volunteering. Low scores indicate that the prospect of meeting people was not an important reason for them to volunteer.

Protective (Pr) – Describes a situation where a volunteer is volunteering as a means of escaping negative feelings about themselves. High scores indicate that a volunteer may be volunteering to help escape from or forget about negative feelings about him/herself. Low scores indicate that the volunteer is not using volunteering as a means to avoid feeling negatively towards him/herself (Clary, Snyder and Ridge, 1992).

Reactivity (Rc) – Describes a situation where a volunteer is volunteering out of a need to heal or address their own past issues. High scores on this scale may indicate that a need to 'right a wrong' in their lives is motivating them to do the volunteer work. Low scores indicate that there is little need for the volunteer to address his or her own past issues through volunteering.

Of these 10 scales, four scales are minor wording adaptations of the work of Clary, Snyder and their colleagues, these being Values, Understanding, Protective and Social. Two further scales being Career Development and Self-Esteem have similarities to the scales developed by Clary, Developing the Volunteer Motivation Inventory, Snyder and their colleagues but contain different statements. The final four scales being Reciprocity, Recognition, Reactivity and Social Interaction are unique scales for Esmond and Dunlop´s VMI.
