# Supplementary material for: Characteristics and motivational factors for joining a lay responder system dispatch to out-of-hospital cardiac arrests
Source: Scand J Trauma Resusc Emerg Med. 2022 Mar 24;30:22. doi: 10.1186/s13049-022-01009-1 (PMC8943963; doi:10.1186/s13049-022-01009-1)
Supplement: Supplementary file 3 — Additional file 3. VMI Template. [file 13049_2022_1009_MOESM3_ESM.pdf]

Supplement 3

VMI Template

a modified version of Esmond and Dunlop´s scoring guide of the Volunteer Motivation Inventory [11].

Scoring Guide

						TOTAL	No. of Questions Answered	Average score
Va	1	11	20	29	38		/5	
Rn	2	12	21*		39		/4	
SI		13	22	31			/3	
Rp	4				40		/2	
Rc	5	14	23	32			/4	
SE	6	15	24	33			/4	
So	7	16	25	34	42		/5	
CD	8		26	35			/3	
Un		18	27	36	43		/4	
Pr	10	19	28	37	44		/5	

Scoring Instructions

This modified Volunteer Motivation Inventory (VMI) consists of thirty-nine reasons that one might have for volunteering and participants were asked to indicate, on the five-point scale, the extent to which they agreed or disagreed with each reason as it applied to them. The original VMI consists of forty-four questions, but No 3, 9, 17, 30 and 41 were not applicable on the type of volunteering SMS-lifesaver perform, why these are blackened in the scoring guide and the figure in the Q´s Answered column is changed to reflect total number of questions in each category.

For each individual, ten scores are calculated that correspond to the ten different motivations to volunteer that are assessed by this inventory. The highest scale score reflects the motivation of greatest importance to the participant while the lowest score reflects the motivation of least concern. When these scale scores are obtained, a manager of volunteers will be able to identify and rank order what are the most important motivation(s) for that particular volunteer.

Step 1. Enter the responses as numbers in order down the columns (the question numbers are written in small text as a guide). Allow some space for corrections in each box. Where an answer is not provided for a question, leave the square blank.

Step 2. Question **21**, marked with an asterisk (*) must be *recoded*. To *recode* this question simply change all 1 responses into 5, 2 responses into 4, 4 responses into 2, and 5 responses into 1. Be sure to cross out the original response, leaving only the recoded response.

Step 3. Add the numbers up in their respective rows and write the total score in the TOTAL column. In the No. Q’s Answered column, write the number of questions that have an answer for each row. In most cases this will be equal to the maximum number of answers, which is specified in that column.

Step 4. Divide the figure in the TOTAL column by the figure in the Q’s Answered column, and write this number in the Average Score column. Repeat this procedure for each row.
