# Supplementary material for: Characteristics and motivational factors for joining a lay responder system dispatch to out-of-hospital cardiac arrests
Source: Scand J Trauma Resusc Emerg Med. 2022 Mar 24;30:22. doi: 10.1186/s13049-022-01009-1 (PMC8943963; doi:10.1186/s13049-022-01009-1)
Supplement: Supplementary file 4 — Additional file 4. Approval from the originators. [file 13049_2022_1009_MOESM4_ESM.pdf]

Supplement 4

Approval from the originators

Following text is email correspondence between developer of Volunteer Motivation Inventory (VMI) and researcher of this project 26.09.2017.

Hi, from Sweden, I have also written to your colleague J Esmond with the same question.

My name is Åsa Högstedt, Gothenburg University. I´m in my very first step in research and working on a PhD. I have been working as a project manager for two years with SMSlifesaver, a system where volunteers, educated in CPR, gets alarmed by positioning of their phones if they are close to a cardiac arrest. It is the dispatch center, who sends out the common response, ambulance service and firebrigade – and in this project also SMSlifesavers.

One of the hypothesis is that it could increase the survival rate, especially in rural areas.
If interested, please read more:
http://www.smslivraddare.se/english/

My first research will try to find out the motivation behind these volunteers.
I have search for validated questionnaires to use and found your interesting project;

“Developing the Volunteer Motivation Inventory to
Assess the Underlying Motivational Drives
of Volunteers in Western Australia”

This is the only questionnaire that I found for weeks, that will fit the type of volunteers I plan to investigate. It covers all the parts that I´m interested in to look deeper into.

Is it possible for me to use your questionnaire and the instrument for calculating data? If so – my research team (Prof. Eric Carlström, PhD Andreas Claesson and PhD Peter Lundgren) propose that I should translate it to Swedish and then back to English to validate if the proper aim of the questions still are the same. We plan to get 20 people to validate with a CVI – Content Validity Index (Polit, 2007) both the translation to Swedish and the translation back to English.

It would be a tremendous help and validation if you could respond to the translation, in that case.

Best regards

Med Vänlig Hälsning
Åsa Högstedt

Hi Åsa,
Thanks for getting in touch! I am very happy for you to use the questionnaire and translate it. I’ve attached a report that might be useful. I’ll also let Judy know that I’ve replied to you; if she has any issues, I’ll ask her to write to you directly.

I’m also thinking that it would be nice to have a translated version of the VMI hosted on the web somewhere alongside the English version, so at some point I might ask if you’d mind sharing the Swedish version. Indeed, I’ve had several enquiries from people from all over the world so I imagine there are versions of it in many different languages. Anyway, that’s a matter for Judy and I to work out…

I’ve also attached the Clary et al. paper which we also found very helpful. Both our instrument and the Clary one are getting a bit old, so you might also consider running a qualitative study to see if there are any contemporary factors that might be attractive to volunteers. E.g., in Australia, we’re finding that the younger generation of volunteers are looking for ‘the experience’ (e.g., travelling to Cambodia to help build something)

rather than the traditional volunteer ‘career’ (e.g., being a volunteer fire fighter for 30+ years). I am not sure whether our instrument would capture these particular motivations well.

Anyway, I hope this is helpful; best of luck with your research project!

Cheers,
Patrick

Dr Patrick Dunlop | Senior Lecturer | School of Psychological Science (M304)
Program Director – Master of Industrial and Organisational Psychology
The University of Western Australia | 35 Stirling Highway, Crawley WA 6009, AUSTRALIA
P +61 8 6488 7614 | patrick.dunlop@uwa.edu.au | www.uwa.edu.au/people/patrick.dunlop
