# Supplementary material for: Characteristics and motivational factors for joining a lay responder system dispatch to out-of-hospital cardiac arrests
Source: Scand J Trauma Resusc Emerg Med. 2022 Mar 24;30:22. doi: 10.1186/s13049-022-01009-1 (PMC8943963; doi:10.1186/s13049-022-01009-1)
Supplement: Supplementary file 5 — Additional file 5. Cluster analysis. [file 13049_2022_1009_MOESM5_ESM.docx]

**Supplement 5 – Cluster analysis**

The lay volunteers were also clustered based on their score for each motivational category using an agglomerative hierarchical clustering method. This analysis grouped volunteers with similar scores for each motivation category together into so-called clusters. The purpose of the cluster analysis was to examine whether there were groups with a different motivation that were otherwise missed by the mean of the entire sample population.

The Elbow method was used to calculate the optimal number of clusters. Each cluster was then summarized based on the mean score for each motivation category along with standard deviation and descriptive data.

**Stratification of the lay responders**

Stratification based on age (Figure 1) showed that with increasing age, the mean scores decreased for the motivation categories *career development*, *self-esteem,* and *understanding*.

Stratifying based on level of education (Figure 2) showed that *career development*, *recognition, self-esteem, social interaction, social, understanding,* and *values* had higher mean scores for volunteers that had completed secondary education.

Stratifying based on gender showed no larger differences between the genders for each motivation category (Figure 3).

**Hierarchical clustering of the lay responders**

The hierarchical cluster analysis was based on the individual scores for motivation categories where, based on the Elbow method, the optimal number of clusters were four. The mean score, standard deviation, and ranking for each motivation category in each cluster are presented in Table 3. The characteristics of each cluster are presented in Table 4.

***Cluster 1***
Cluster one was the largest cluster (n = 242). This cluster had a highest mean score for *values* (M 3.97; SD 0.44). *Social interaction* (M 1.81; SD 0.63) was ranked ninth out of ten and *career development* was ranked sixth out of ten (M 2.27; SD 0.71). The ranking of the remaining motivation categories did not differ greatly from the entire study population or the other clusters.
This cluster consisted of 61% female volunteers and a majority of the volunteers were in the age group 25-39 years (51.2%). A majority of the volunteers had completed post-secondary education (70.6%).

***Cluster 2***
Cluster two was the second largest cluster (n = 122). This cluster had a highest mean score for *values* (M 3.65; SD 0.63). *Social interaction* (M 1.46; SD 0.52) was ranked eighth out of ten and *career development* (M 1.35; SD 0.39) was ranked ninth out of ten. The ranking of the remaining motivation categories did not differ greatly from the entire study population or the other clusters.
This cluster consisted of 58% female volunteers and a majority of the volunteers were in the age groups 25-39 and 40-54 years (40.2% and 36.9%, respectively). A majority of the volunteers had completed post-secondary education (73.7%).

***Cluster 3***
Cluster three was the second smallest cluster (n= 75). This cluster had a highest mean score for Reciprocity (M 4.33; SD 0.74). *Social* *interaction* was ranked sixth out of ten (M 2.94; SD 0.67) and *career development* was ranked seventh out of ten (M 2.92; SD 0.58). The ranking of the remaining motivation categories did not differ greatly from the entire study population or the other clusters.
This cluster consisted of 55% female volunteers and a majority of the volunteers were in the age groups 25-39 and 40-54 years (32.0% and 34.7%, respectively). A large proportion of the volunteers had completed secondary education (49.4%).

***Cluster 4***
Cluster four was the smallest cluster (n = 22). This cluster had a highest mean score for *reciprocity* (M 4.70; SD 0.43). *Career development* was ranked sixth out of ten (M 4.03; SD 0.49) and *social interaction* was ranked seventh out of ten (M 4.00; SD 0.58).
This cluster consisted of 46% female volunteers and a majority of the volunteers were in the age group 25-39 years (54.5%). A large proportion of the volunteers had completed secondary education (68.2%).

In all four clusters, the motivational categories *values, reciprocity, self-esteem,* r*ecognition,* and *understanding* were ranked in the top five. Similarly, these motivational categories were ranked in the top five in the overall study population.

**Stratification findings**

The decreasing mean scores for *career development* with each increasing age group in the stratification based on age (Figure 1) suggests that this motivation becomes less important with increasing age. Note that the mean scores for certain motivational categories such as *career development* and *understanding* where higher for the oldest age groups, 65 years and older. These results must be interpreted with caution as these volunteers only made up 2.4% of the study population. Intuitively, young volunteers that are either students or in the early stages of their careers are likely to value the experiences that volunteering will yield their careers. A similar pattern for *career development* was presented in Ho, You and Fung’s research (35); the authors described younger volunteers as more motivated to improve their future employment prospects than older volunteers. Additionally, greater importance for *understanding* in the younger volunteers suggests that these volunteers are motivated to learn through the experiences from volunteering. A large proportion of the volunteers were also healthcare professionals. Therefore, *career development* and *understanding* are likely strong motivators amongst the younger volunteers with careers in healthcare.

*Self-esteem* was also a stronger motivator for younger volunteers which corresponds with Cho, Bonn and Han’s research (36). These results suggest that the younger volunteers are in part motivated to become first responders with the expectation that the experiences will elevate their self-worth. Here again, the higher means score of the age group 65-74 years is possibly misleading.

*Social interaction* was valued more in the older age groups. However, there were very few volunteers in these age groups, thus these results may not be representative of a greater population.

**Findings from the cluster analysis**

With consideration to the high ranking of both *values* and *reciprocity,* the result exemplifies how the act of volunteering is in itself only interesting for individuals with motivations with largely intrinsic components, as seen in previous studies (11, 26-31).

However, the mean scores and ranking of individual motivation categories did vary slightly between the clusters, primarily in the categories ranked below fifth in importance. The background characteristics of the volunteers in each cluster was examined for observable patterns that could explain these differences. For example, clusters two and three had a larger proportion of volunteers in the age group 40 – 54 years compared to clusters one and four, which had a larger proportion in the age group 25 – 39. This could explain why *career development* had lower rankings in clusters two and three with more older volunteers. These results also support the previously mentioned findings from the stratification based on age. Thus, emphasis on the impact that volunteering may have on professional and career development may be of value in the recruitment of younger volunteers.

Additionally, clusters three and four had higher mean scores and rankings for *reciprocity* and *social interaction*. The volunteers in these clusters were more likely to have completed secondary education, as opposed to a larger proportion of volunteers with post-secondary education in clusters one and two. In the stratification of the scores for each motivation category based on level of education, it was shown that volunteers with secondary education scored *social interaction* as more important. However, the score for *reciprocity* was not influenced by level of education. Thus, solely *social interaction* may be associated with level of education.

**Table 3**: The ranking of motivation categories for each cluster based on mean scores. Mean (standard deviation).

| **Most important**  **Least important** | **Overall** | **Cluster 1** | **Cluster 2** | **Cluster 3** | **Cluster 4** |
| --- | --- | --- | --- | --- | --- |
|  | Values  3.97 (0.55) | Values  3.97 (0.44) | Values  3.65 (0.63) | Reciprocity  4.33 (0.74) | Reciprocity  4.70 (0.43) |
|  | Reciprocity  3.88 (0.9) | Reciprocity  3.94 (0.75) | Reciprocity  3.34 (1.0) | Values  4.29 (0.49) | Self-esteem  4.56 (0.47) |
|  | Self-esteem  3.22 (0.92) | Self-esteem  3.30 (0.7) | Self-esteem  2.31 (0.56) | Self-esteem  4.08 (0.67) | Values  4.55 (0.42) |
|  | Recognition  2.76 (0.77) | Understanding  2.84 (0.86) | Recognition  2.29 (0.59) | Understanding  3.70 (0.61) | Understanding  4.45 (0.53) |
|  | Understanding  2.74 (1.08) | Recognition  2.70 (0.66) | Understanding  1.63 (0.55) | Recognition  3.32 (0.67) | Recognition  4.06 (0.51) |
|  | Career development  2.22 (0.9) | Career development  2.27 (0.71) | Social  1.56 (0.52) | Social interaction  2.94 (0.67) | Career development  4.03 (0.49) |
|  | Social  2.04 (0.8) | Social  1.89 (0.6) | Protective  1.49 (0.31) | Career development  2.92 (0.58) | Social interaction  4.00 (0.58) |
|  | Social interaction  2.01 (0.89) | Protective  1.82 (0.36) | Social interaction  1.46 (0.52) | Social  2.79 (0.69) | Reactivity  3.73 (0.63) |
|  | Protective  1.93 (0.64) | Social interaction  1.81 (0.63) | Career development  1.35 (0.39) | Protective  2.53 (0.67) | Social  3.67 (0.48) |
|  | Reactivity  1.65 (0.79) | Reactivity  1.46 (0.5) | Reactivity  1.23 (0.38) | Reactivity  2.35 (0.76) | Protective  3.45 (0.67) |

Higher score reflects motivation of greater importance to the participant, i.e. more self-determined, and the lower score represent motivation of less importance, i.e. less self-determined. Three is considered neutral.

**Table 4**: Characteristics of the lay volunteers in each cluster

|  | **Cluster 1** **n = 242** | **Cluster 2** **n = 122** | **Cluster 3** **n = 75** | **Cluster 4** **n = 22** |
| --- | --- | --- | --- | --- |
| ***Gender (%)*** | | | | |
| Female | 147 (60.7) | 71 (58.2) | 41 (54.7) | 10 (45.5) |
| ***Age (%)*** | | | | |
| 18-24 years | 41 (16.9) | 6 (4.9) | 14 (18.7) | 5 (22.7) |
| 25-39 years | 124 (51.2) | 49 (40.2) | 24 (32.0) | 12 (54.5) |
| 40-54 years | 60 (24.8) | 45 (36.9) | 26 (34.7) | 3 (13.6) |
| 55-64 years | 14 (5.8) | 19 (15.6) | 7 (9.3) | 1 (4.5) |
| 65-74 years | 3 (1.2) | 2 (1.6) | 3 (4.0) | 1 (4.5) |
| 75 years or older | 0 (0.0) | 1 (0.8) | 1 (1.3) | 0 (0.0) |
| ***Education (%)*** | | | | |
| Primary school < 9 years | 0 (0.0) | 1 (0.8) | 0 (0.0) | 0 (0.0) |
| Primary school, ≥ 9 years | 5 (2.1) | 3 (2.5) | 3 (4.0) | 0 (0.0) |
| Secondary school, ≤ 2 years | 16 (6.6) | 9 (7.4) | 8 (10.7) | 2 (9.1) |
| Secondary school, 3 years | 50 (20.7) | 19 (15.6) | 29 (38.7) | 13 (59.1) |
| Post-secondary education, < 3 years | 56 (23.1) | 21 (17.2) | 12 (16.0) | 3 (13.6) |
| Post-secondary education, ≥ 3 years | 113 (46.7) | 63 (51.6) | 23 (30.7) | 3 (13.6) |
| Postgraduate education | 2 (0.8) | 6 (4.9) | 0 (0.0) | 1 (4.5) |
| ***Occupational area (%)*** | | | | |
| Healthcare | 88 (36.4) | 49 (40.2) | 25 (33.3) | 6 (27.3) |
| Student | 44 (18.2) | 9 (7.4) | 15 (20.0) | 3 (13.6) |
| Police or fire services | 17 (7.0) | 15 (12.3) | 8 (10.7) | 3 (13.6) |
| Teacher | 14 (5.8) | 8 (6.6) | 1 (1.3) | 0 (0.0) |
| EMS | 5 (2.1) | 5 (4.1) | 2 (2.7) | 1 (4.5) |
| Prison guard/watchman | 7 (2.9) | 1 (0.8) | 3 (4.0) | 1 (4.5) |
| Retired | 2 (0.8) | 4 (3.3) | 4 (5.3) | 1 (4.5) |
| Engineer | 2 (0.8) | 3 (2.5) | 0 (0.0) | 0 (0.0) |
| Other occupation | 63 (26.0) | 28 (23.0) | 17 (22.7) | 7 (31.8) |
| ***Motivation Category (Mean (SD))*** | | | | |
| Values | 3.97 (0.43) | 3.65 (0.63) | 4.29 (0.48) | 4.55 (0.41) |
| Reciprocity | 3.94 (0.75) | 3.34 (1.03) | 4.33 (0.74) | 4.70 (0.43) |
| Self-esteem | 3.30 (0.70) | 2.31 (0.56) | 4.08 (0.67) | 4.56 (0.47) |
| Recognition | 2.70 (0.66) | 2.29 (0.59) | 3.32 (0.67) | 4.06 (0.51) |
| Understanding | 2.84 (0.86) | 1.63 (0.55) | 3.70 (0.61) | 4.45 (0.53) |
| Career development | 2.27 (0.71) | 1.35 (0.39) | 2.92 (0.58) | 4.03 (0.49) |
| Social | 1.89 (0.60) | 1.56 (0.52) | 2.79 (0.69) | 3.67 (0.48) |
| Social interaction | 1.81 (0.63) | 1.46 (0.52) | 2.94 (0.67) | 4.00 (0.58) |
| Protective | 1.82 (0.36) | 1.49 (0.31) | 2.53 (0.67) | 3.45 (0.67) |
| Reactivity | 1.46 (0.50) | 1.23 (0.38) | 2.35 (0.76) | 3.73 (0.63) |

**Table 5**: Other Occupations

| **All with Other occupations,** n =115 | |
| --- | --- |
| ***Gender (%)*** | |
| Female | 41 (35.7) |
| ***Age (%)*** | |
| 18-24 years | 10 (8.7) |
| 25-39 years | 50 (43.5) |
| 40-54 years | 44 (38.3) |
| 55-64 years | 10 (8.7) |
| 65-74 years | 1 (0.9) |
| ***Education (%)*** | |
| Primary school, < 9 years | 0 (0.0) |
| Primary school, ≥9 years | 4 (3.5) |
| Secondary school, ≤ 2 years | 11 (9.6) |
| Secondary school, 3 years | 37 (32.2) |
| Post-secondary education, <3 years | 25 (21.7) |
| Post-secondary education, ≥3 years | 35 (30.4) |
| Postgraduate education | 3 (2.6) |
| ***Motivation Categories*** | |
| RP (mean (SD)) | 4.03 (0.90) |
| Va (mean (SD)) | 3.86 (0.56) |
| SE (mean (SD)) | 3.28 (0.89) |
| UN (mean (SD)) | 2.83 (1.04) |
| RN (mean (SD)) | 2.80 (0.74) |
| CD (mean (SD)) | 2.14 (0.80) |
| SI (mean (SD)) | 2.10 (0.93) |
| SO (mean (SD)) | 2.10 (0.76) |
| PR (mean (SD)) | 1.97 (0.70) |
| RC (mean (SD)) | 1.70 (0.84) |


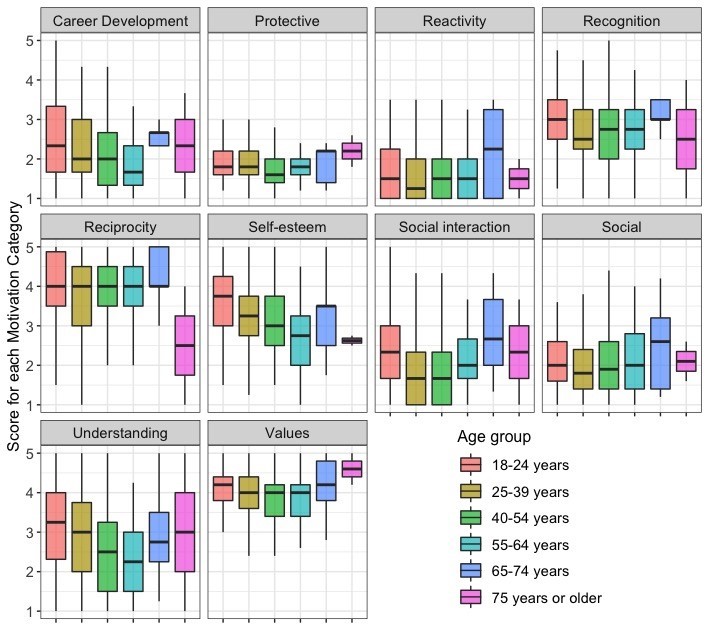


**Figure 1**: Scores for each motivation category stratified by age group


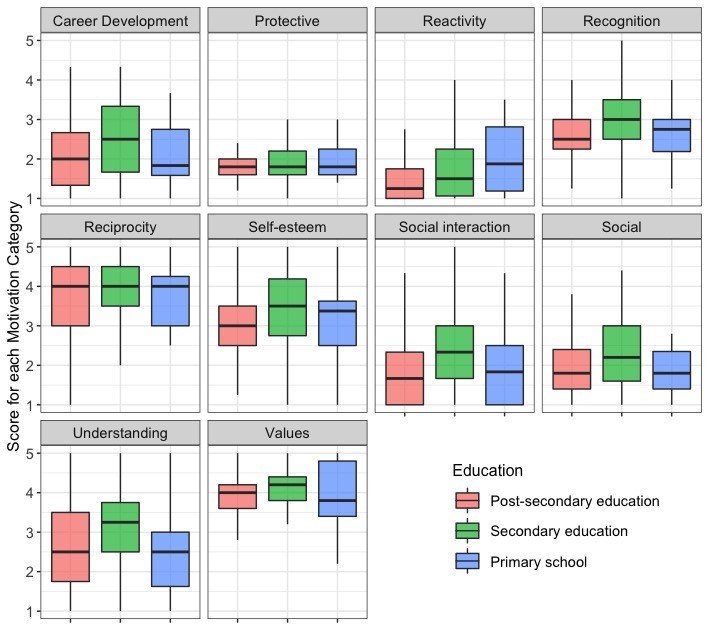


**Figure 2:** Scores for each motivation category stratified by education


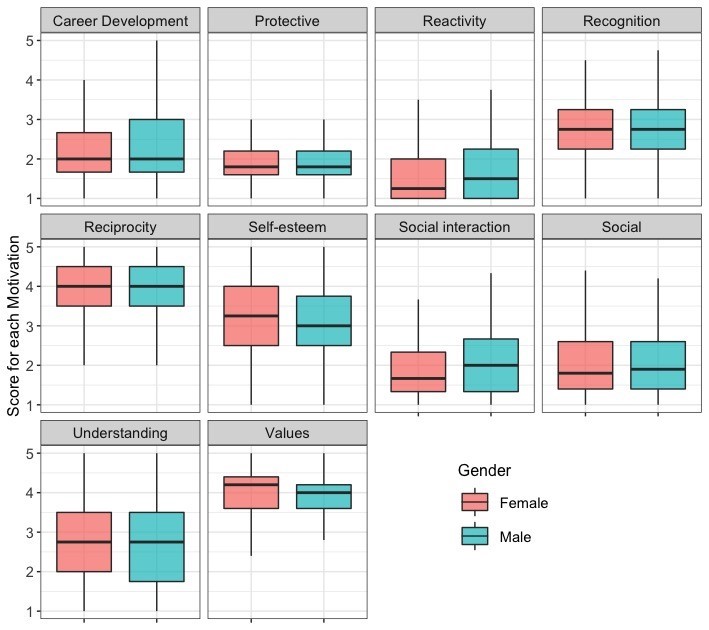


**Figure 3**: Score for each motivation category stratified by gender
